# Botulinum toxin type A for myogenic temporomandibular disorders: A scoping review of injection protocols, clinical outcomes, safety, and evidence gaps

**DOI:** 10.1016/j.jobcr.2026.101556

**Published:** 2026-10-12

**Authors:** Marines Vega Sanchez, Luis Chauca Bajaña, Francisco Córdova, Maria Rodriguez Tates, Byron Velasquez Ron

**Affiliations:** a Carrera de Odontología, Facultad de Odontología, Universidad de Las Américas Ecuador (UDLA), Colon y 6 de Diciembre, Quito, 170516, Ecuador; b School of Dentistry, Universidad Bolivariana del Ecuador (Durán ), Guayaquil, 090101, Ecuador

**Keywords:** Botulinum toxin type A, Temporomandibular disorders, Myofascial pain, Myalgia, Masticatory muscles, Pain, Muscle function, Safety

## Abstract

**Background:**

Botulinum toxin type A (BoNT-A) has been increasingly investigated for persistent myogenic temporomandibular disorders (TMDs), particularly when conservative therapies provide insufficient symptom control. However, variability in patient selection, injection protocols, outcomes, and safety reporting complicates interpretation of its effectiveness.

**Objective:**

To map and critically synthesize evidence regarding BoNT-A injection protocols, clinical outcomes, functional and physiological effects, safety, and methodological limitations in adults with myogenic TMD.

**Methods:**

This scoping review followed PRISMA-ScR. PubMed/MEDLINE, Scopus, Web of Science Core Collection, and Embase were searched from inception to August 2026. Clinical studies involving adults with myogenic TMD, masticatory myalgia, or myofascial pain treated with intramuscular BoNT-A were included. Data on injection protocols, pain, function, physiological effects, and safety were charted, and risk of bias was appraised using design-appropriate tools.

**Results:**

Of 1470 records identified, 770 remained after duplicate removal, and 20 clinical studies were included. Substantial heterogeneity existed in diagnostic criteria, formulation, dose, targeted muscles, injection techniques, comparators, outcomes, and follow-up. Pain and patient-reported outcomes frequently improved, particularly in uncontrolled studies, whereas placebo-controlled trials showed less consistent superiority. Pain improvement did not consistently parallel functional recovery. Objective assessments demonstrated reduced muscle thickness, electromyographic activity, bite force, and masticatory performance. No standardized protocol or optimal dose with an established benefit–risk profile was identified.

**Conclusions:**

BoNT-A may provide symptomatic benefit in selected patients with persistent myogenic TMD, but evidence does not support routine first-line use. Standardized protocols, multidimensional outcomes, and longer-term randomized trials are needed.

## 1 Introduction

Temporomandibular disorders (TMDs) comprise a heterogeneous group of musculoskeletal conditions affecting the temporomandibular joints, masticatory muscles, and associated structures. Among these conditions, myogenic TMDs, including masticatory myalgia and myofascial pain, represent a frequent source of persistent orofacial pain and may substantially impair mandibular function, oral health-related quality of life, and daily activities.^1^ Their multifactorial nature and the interaction between peripheral nociceptive mechanisms, muscle dysfunction, psychosocial factors, and pain sensitization frequently complicate clinical management.^2^ Current management of myogenic TMD is primarily conservative and may include patient education, behavioral modification, pharmacological therapy, physiotherapy, therapeutic exercises, and occlusal appliances. Although these approaches provide satisfactory symptom control in many patients, a subgroup develops persistent or recurrent pain despite conventional treatment. This therapeutic challenge has stimulated interest in adjunctive interventions capable of modulating both muscular activity and nociceptive signaling. Botulinum toxin type A (BoNT-A) has consequently emerged as a potential therapeutic option for persistent myogenic TMD. By inhibiting acetylcholine release at the neuromuscular junction, BoNT-A produces a temporary reduction in muscle contractility. Experimental and clinical evidence also suggests effects on peripheral nociceptive pathways through modulation of pain-related neurotransmitter release.^3^ These mechanisms provide a biological rationale for its use in patients with painful hyperactivity of the masticatory muscles or persistent myofascial pain. Clinical studies, however, have produced heterogeneous findings. Several observational investigations and uncontrolled clinical studies have reported reductions in pain intensity and improvements in patient-reported quality of life after BoNT-A administration. In contrast, randomized placebo-controlled trials have not consistently demonstrated clinically meaningful superiority over placebo or conservative therapies. Moreover, substantial variability exists across studies regarding patient selection, diagnostic criteria, BoNT-A formulation, total dose, dose per muscle, muscles targeted, number and location of injection sites, injection guidance, comparator interventions, outcome measures, and duration of follow-up. Safety represents an additional area of clinical uncertainty.^4^ Although BoNT-A is generally considered well tolerated, its therapeutic mechanism necessarily involves a reduction in masticatory muscle activity. Recent clinical investigations have documented decreases in muscle thickness, electromyographic activity, and bite force after injection, while other studies have raised concerns regarding dose-dependent changes in masticatory performance and mandibular bone parameters. Importantly, the magnitude and duration of these physiological effects do not necessarily parallel pain relief, emphasizing the need to evaluate efficacy and safety simultaneously rather than considering analgesic outcomes in isolation.^5^ Previous systematic reviews and meta-analyses have primarily addressed whether BoNT-A reduces pain in TMD or bruxism-related conditions. However, differences in diagnostic populations, intervention protocols, comparators, and outcome definitions have limited the clinical interpretability of pooled estimates.^6^ Furthermore, the increasing number of recent randomized trials, prospective cohorts, and studies specifically examining muscular and functional consequences provides an opportunity to reassess the field from a broader clinical perspective.^7^ A comprehensive mapping of how BoNT-A is currently administered, which outcomes have been investigated, what safety signals have been identified, and where methodological inconsistencies persist may therefore be more informative than focusing exclusively on a pooled estimate of analgesic efficacy.^8^Accordingly, this scoping review aimed to map and critically synthesize the available clinical evidence on BoNT-A for myogenic TMD, with particular emphasis on injection protocols, therapeutic outcomes, functional and physiological effects, safety, and methodological quality. 8, 9 A secondary objective was to identify sources of clinical and methodological heterogeneity and current evidence gaps that limit protocol standardization and the development of evidence-based recommendations for routine clinical practice.

## 2 Materials and methods

### 2.1 Study design and reporting framework

This scoping review was conducted to systematically map the clinical evidence regarding the use of botulinum toxin type A (BoNT-A) for the management of myogenic temporomandibular disorders (TMDs). The review was structured according to the methodological framework for scoping reviews and reported in accordance with the Preferred Reporting Items for Systematic Reviews and Meta-Analyses Extension for Scoping Reviews (PRISMA-ScR). The study-selection process was documented using a PRISMA-style flow diagram to ensure transparent reporting of record identification, screening, eligibility assessment, and final inclusion.^10^ The review was designed to characterize the available evidence rather than to estimate a single pooled treatment effect. Particular emphasis was placed on differences in patient selection, diagnostic criteria, BoNT-A formulations and injection protocols, therapeutic outcomes, functional and physiological effects, safety findings, and methodological limitations.

### 2.2 Review question and PCC framework

The review question was formulated according to the Population–Concept–Context (PCC) framework recommended for scoping reviews. The Population comprised adults aged 18 years or older with myogenic TMD, masticatory muscle myalgia, or masticatory myofascial pain. Studies including concomitant conditions such as bruxism were considered eligible only when myogenic TMD or masticatory myofascial pain was explicitly identified as part of the clinical diagnosis and the relevant outcomes could be attributed to the target population.^11^ The Concept was the therapeutic administration of intramuscular BoNT-A to the masticatory muscles, either as a stand-alone intervention or in comparison with placebo, conservative treatment, occlusal appliances, pharmacological interventions, physical/manual therapies, or alternative injection protocols.^12^ The Context included dental, oral and maxillofacial, orofacial pain, pain medicine, and multidisciplinary clinical settings in which BoNT-A was administered for the management of myogenic TMD.

Accordingly, the primary review question was:

What clinical evidence is available regarding BoNT-A injection protocols, therapeutic outcomes, functional and physiological effects, and safety in adults with myogenic temporomandibular disorders?

A secondary question addressed which methodological and clinical sources of heterogeneity currently limit standardization of BoNT-A protocols and evidence-based recommendations for routine clinical practice.

### 2.3 Eligibility criteria

Original clinical studies were considered eligible when they fulfilled the following criteria: (1) participants were adults aged ≥18 years; (2) the study population had a diagnosis of myogenic TMD, masticatory myalgia, or masticatory myofascial pain based on the Diagnostic Criteria for Temporomandibular Disorders (DC/TMD), Research Diagnostic Criteria for Temporomandibular Disorders (RDC/TMD), or clearly defined equivalent clinical diagnostic criteria^1^; (3) BoNT-A was administered intramuscularly to one or more masticatory muscles; and (4) the study reported at least one clinically relevant outcome related to pain, mandibular function, quality of life, muscle activity or morphology, bite force, treatment response, or adverse effects. Randomized controlled trials, non-randomized controlled clinical studies, prospective and retrospective cohort studies, before-and-after studies, and prospective clinical pilot studies were eligible.^12^ Studies were excluded when they evaluated BoNT-A exclusively for sleep bruxism without a defined myogenic TMD diagnosis, obstructive sleep apnea, cosmetic masseter hypertrophy, isolated headache disorders, or exclusively arthrogenic TMD. Studies involving pediatric or adolescent populations were excluded unless data for participants aged ≥18 years could be separately extracted. Animal and in vitro studies, case reports, narrative or systematic reviews, meta-analyses, editorials, letters, conference abstracts without sufficient clinical data, and studies without extractable outcomes relevant to myogenic TMD were also excluded. Studies including bruxism as a comorbidity were not automatically excluded when a diagnosis of masticatory myofascial pain or myogenic TMD was explicitly established.

### 2.4 Information sources and search strategy

A comprehensive electronic search was performed in PubMed/MEDLINE, Scopus, Web of Science Core Collection, and Embase from database inception to August 8, 2026. The search strategy combined controlled vocabulary terms, where available, with free-text terms related to temporomandibular disorders, masticatory myalgia, myofascial pain, and botulinum toxin type A. The PubMed/MEDLINE strategy was adapted to the syntax and indexing requirements of each database. No restrictions on publication year were applied. Reference lists of included studies and relevant systematic reviews were additionally screened to identify potentially eligible studies not retrieved through the electronic database search. Forward citation tracking of key eligible studies was also undertaken to identify recently published evidence. The complete database-specific search strategies, including controlled vocabulary, free-text terms, Boolean operators, date of search, and number of records retrieved, are provided in Table 1.

**Table 1:** Data base specific strategies.

PUBMED	SCOPUS	WEB SCIENCE	EMBASE
(“Temporomandibular Joint Disorders" [Mesh] OR “temporomandibular disorder*" [Title/Abstract] OR “temporomandibular dysfunction*" [Title/Abstract] OR “myogenous temporomandibular disorder*" [Title/Abstract] OR “myogenic temporomandibular disorder*" [Title/Abstract] OR “masticatory myalgia" [Title/Abstract] OR “masticatory muscle pain" [Title/Abstract] OR “masticatory myofascial pain" [Title/Abstract] OR “myofascial pain" [Title/Abstract] OR “orofacial myalgia" [Title/Abstract]) AND (“Botulinum Toxins, Type A" [Mesh] OR “botulinum toxin type A" [Title/Abstract] OR “botulinum toxin A" [Title/Abstract] OR “BoNT-A" [Title/Abstract] OR “BoNT/A" [Title/Abstract] OR onabotulinumtoxin A [Title/Abstract] OR incobotulinumtoxinA [Title/Abstract] OR abobotulinumtoxin A [Title/Abstract] OR Botox [Title/Abstract] OR Xeomin [Title/Abstract] OR Dysport [Title/Abstract])	TITLE-ABS-KEY (“temporomandibular disorder*" OR “temporomandibular dysfunction*" OR “myogenous temporomandibular disorder*"OR “myogenic temporomandibular disorder*"OR “masticatory myalgia” OR “masticatory muscle pain"OR “masticatory myofascial pain” OR “myofascial pain"OR “orofacial myalgia”) AND TITLE-ABS-KEY (“botulinum toxin type A"OR “botulinum toxin A″ OR “BoNT-A″ OR “BoNT/A″ OR onabotulinumtoxin A OR incobotulinumtoxin A OR abobotulinumtoxin A OR Botox OR Xeomin OR Dysport**)**	TS=(“temporomandibular disorder*"OR “temporomandibular dysfunction*" OR “myogenous temporomandibular disorder*"OR “myogenic temporomandibular disorder* OR “masticatory myalgia” OR “masticatory muscle pain"OR “masticatory myofascial pain"OR “myofascial pain OR “orofacial myalgia”) AND TS=(“botulinum toxin type A"OR “botulinum toxin A″ OR “BoNT-A"OR “BoNT/A"OR onabotulinumtoxin A OR incobotulinumtoxin A OR abobotulinumtoxin A OR Botox OR Xeomin OR Dysport)	(‘temporomandibular joint disorder'/exp OR ‘temporomandibular disorder*':ti,ab,kw OR ‘myogenous temporomandibular disorder*':ti,ab,kw OR ‘myogenic temporomandibular disorder*':ti,ab,kw OR ‘masticatory myalgia':ti,ab,kw OR ‘masticatory muscle pain':ti,ab,kw OR ‘masticatory myofascial pain':ti,ab,kw OR ‘myofascial pain':ti,ab,kw OR ‘orofacial myalgia':ti,ab,kw) AND (‘botulinum toxin a'/exp OR ‘botulinum toxin type A':ti,ab,kw OR ‘botulinum toxin A':ti,ab,kw OR ‘BoNT-A':ti,ab,kw OR ‘onabotulinumtoxinA':ti,ab,kw OR ‘incobotulinumtoxinA':ti,ab,kw OR ‘abobotulinumtoxinA':ti,ab,kw OR ‘Botox':ti,ab,kw OR ‘Xeomin':ti,ab,kw OR ‘Dysport':ti,ab,kw)

### 2.5 Study selection

After removal of duplicate records, titles and abstracts were screened against the predefined eligibility criteria. Potentially relevant articles were subsequently retrieved in full text and independently assessed for eligibility. Studies that did not fulfill the inclusion criteria were excluded at the full-text stage, and the primary reason for exclusion was recorded. Reasons included inappropriate population, absence of a defined myogenic TMD diagnosis, nonclinical study design, inappropriate intervention, absence of relevant clinical outcomes, and noneligible publication type.^13^ Disagreements regarding eligibility were resolved through discussion and consensus between reviewers. When necessary, a third reviewer was consulted.Multiple publications originating from the same trial or patient cohort were identified by comparing authorship, recruitment periods, sample characteristics, intervention protocols, trial registration information, and study settings. Such publications were treated as multiple reports of a single underlying study rather than as independent studies whenever cohort overlap was established. The complete selection process will be presented in the PRISMA flow diagram (Fig. 1).

**Fig. 1:**
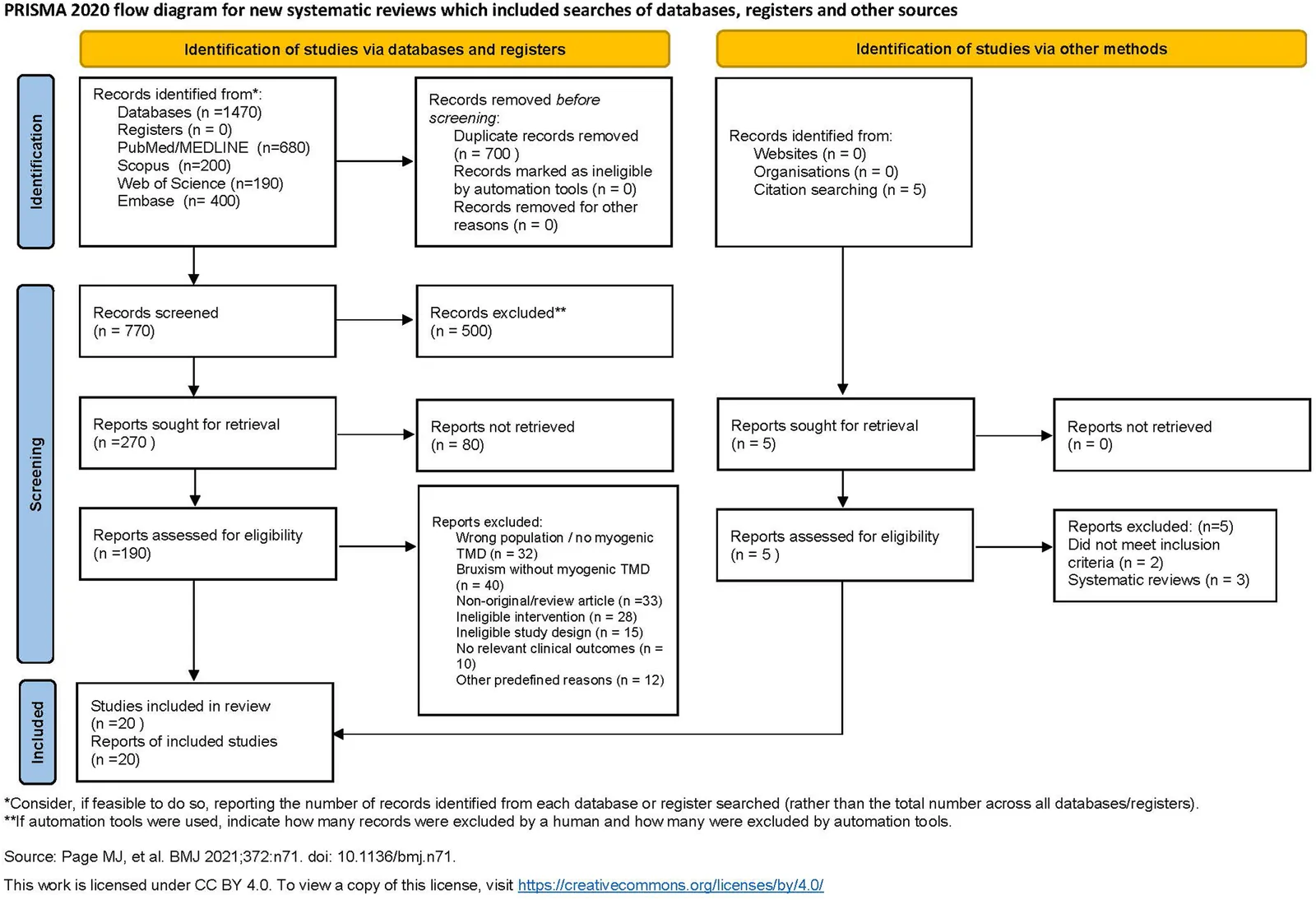
**PRISMA 2020 flow diagram of the study-selection process.** Records were identified through PubMed/MEDLINE, Scopus, Web of Science Core Collection, and Embase. Additional reports were identified through citation searching. After duplicate removal, title/abstract screening, full-text assessment, and application of the predefined eligibility criteria, 20 independent studies were included in the review.

### 2.6 Data extraction and data charting

Data were systematically charted using a standardized extraction form developed specifically for this review. The extraction framework was designed to capture both conventional study characteristics and clinically relevant variations in BoNT-A administration.The following information was extracted whenever available: author and year of publication, country, study design, sample size, participant age and sex, diagnostic criteria, clinical TMD phenotype, duration of symptoms, BoNT-A formulation, total administered dose, dose per muscle and per side, dilution, muscles injected, number and location of injection sites, injection localization or guidance method, comparator intervention, number of treatment sessions, follow-up duration, pain outcomes, mandibular function, maximum mouth opening, quality-of-life measures, electromyographic outcomes, bite force, muscle morphology or thickness, bone-related outcomes, treatment response, and adverse events. For studies with multiple intervention groups or BoNT-A doses, data were extracted separately for each treatment arm whenever possible. When multiple reports originated from the same underlying study, complementary outcomes were combined at the study level while avoiding duplicate counting of participants. Data extraction was performed independently by two reviewers, and discrepancies were resolved through consensus.

### 2.7 Outcomes and evidence domains

Extracted outcomes were organized into five predefined evidence domains:

(1) **Injection protocols:** BoNT-A formulation, total dose, dose per muscle, targeted muscles, number of injection sites, injection guidance, and treatment frequency.
(2) **Pain-related outcomes:** pain intensity, pressure pain threshold, pain frequency, pain-related disability, and patient-reported treatment response.
(3) **Functional and patient-reported outcomes:** mandibular mobility, maximum mouth opening, jaw functional limitation, masticatory performance, oral health-related quality of life, and general health-related quality of life.
(4) **Physiological and safety outcomes:** adverse events, muscle weakness, electromyographic activity, bite force, muscle thickness or volume, masticatory performance, and mandibular bone-related changes.
(5) **Methodological characteristics and evidence gaps:** study design, diagnostic criteria, comparator selection, follow-up duration, sample size, risk of bias, and reporting completeness.

This domain-based approach was used to facilitate comparison across clinically heterogeneous studies and to identify areas in which protocol standardization remains limited.

### 2.8 Methodological quality and risk-of-bias assessment

Randomized controlled trials were assessed using the Cochrane Risk of Bias 2 (RoB 2) tool across five domains: bias arising from the randomization process, deviations from intended interventions, missing outcome data, measurement of the outcome, and selection of the reported result.^14^ Domain-level judgments were categorized as low risk, some concerns, or high risk, and an overall risk-of-bias judgment was assigned to each trial. Non-randomized studies of interventions were assessed using ROBINS-I across seven domains: confounding, participant selection, classification of interventions, deviations from intended interventions, missing data, outcome measurement, and selection of the reported result. Judgments were categorized as low, moderate, serious, or critical risk of bias. Methodological appraisal was used to inform interpretation of the evidence rather than as an exclusion criterion.^15^

### 2.9 Data synthesis

Given the purpose of this scoping review and the substantial clinical and methodological heterogeneity across the included studies, findings were synthesized descriptively rather than quantitatively pooled. Studies differed substantially in diagnostic criteria, BoNT-A formulations, doses, muscles injected, injection techniques, comparator interventions, outcome measures, and follow-up periods.^16^ Evidence was therefore mapped according to predefined domains, including injection protocols, pain-related outcomes, functional and patient-reported outcomes, physiological and safety effects, and methodological characteristics. Randomized controlled trials were considered separately from observational and uncontrolled studies when interpreting therapeutic outcomes, particularly to distinguish within-group improvement from treatment effects observed against placebo or active comparators.^17^ No meta-analysis was performed because quantitative pooling was not required to address the mapping objectives of the review and the clinical and methodological heterogeneity of the available evidence would have limited the interpretability of a single pooled effect estimate.

### 2.10 Ethical considerations

As this study analyzed data from previously published research and did not involve direct participation of human subjects or access to identifiable individual-level data, institutional ethics committee approval and informed consent were not required.

## 3 Results

### 3.1 Study selection

The systematic search identified 1470 records across the four electronic databases: PubMed/MEDLINE (n = 680), Scopus (n = 200), Web of Science Core Collection (n = 190), and Embase (n = 400). After removal of 700 duplicate records, 770 records remained for title and abstract screening. Of these, 500 were excluded because they did not meet the predefined eligibility criteria, leaving 270 reports for full-text retrieval. Eighty reports could not be retrieved, and 190 full-text reports were therefore assessed for eligibility. Of these, 170 were excluded for the following reasons: wrong population or absence of a defined myogenic TMD diagnosis (n = 32), bruxism without myogenic TMD (n = 40), non-original or review articles (n = 33), ineligible intervention (n = 28), ineligible study design (n = 15), absence of relevant clinical outcomes (n = 10), and other predefined reasons (n = 12). In addition, citation searching identified five potentially relevant reports. All five were successfully retrieved and assessed for eligibility; two did not meet the inclusion criteria and three were systematic reviews. Consequently, none of the reports identified through citation searching contributed additional eligible studies. The final evidence synthesis comprised 20 reports representing 20 independent clinical studies evaluating BoNT-A in adults with myogenic TMD or masticatory myofascial pain. The complete study-selection process is presented in Fig. 1.

### 3.2 Characteristics of the included studies

The included studies encompassed randomized controlled trials, prospective and retrospective observational studies, and pilot clinical investigations evaluating botulinum toxin type A (BoNT-A) for myogenic temporomandibular disorders or masticatory myofascial pain. Considerable methodological heterogeneity was observed across studies with respect to sample size, diagnostic criteria, treatment protocols, comparator interventions, outcome measures, and duration of follow-up.

Earlier studies predominantly evaluated pain-related outcomes, whereas more recent investigations incorporated multidimensional endpoints, including mandibular function, oral health-related quality of life, electromyographic activity, bite force, masticatory performance, muscle morphology, and other physiological outcomes. Diagnostic definitions also varied across the literature. Several studies used the Research Diagnostic Criteria for Temporomandibular Disorders (RDC/TMD) or the Diagnostic Criteria for Temporomandibular Disorders (DC/TMD),^1^ whereas others relied on clinically defined masticatory myofascial pain or myogenous TMD criteria.

Sample sizes varied substantially.^18^ Several studies were small pilot or exploratory investigations, whereas more recent trials and observational cohorts included larger patient populations. For example, Villa et al. evaluated 28 patients with RDC/TMD-defined myofascial pain,^19^ while Park et al. prospectively investigated 15 patients with myogenic TMD.^20^ Van Soest et al. evaluated 35 patients with DC/TMD-defined TMD-related myalgia and followed clinical outcomes for up to 6 months.^21^

The main characteristics of the 20 included studies, including study design, diagnostic criteria, BoNT-A formulation and injection protocol, target muscles, comparators, follow-up periods, outcome measures, and principal findings, are summarized in Table 2.

**Table 2:** Characteristics of clinical studies evaluating botulinum toxin type A in myogenic temporomandibular disorders/masticatory myofascial pain.

Study	Design	Population/diagnostic criteria	N	BoNT-A formulation	Injection protocol	Target muscles/technique	Comparator	Follow-up	Outcomes	Main findings/safety
Nixdorf et al. (2002)^13^	Randomized, double-blind, placebo-controlled crossover trial	15 women aged 18–45 years with chronic moderate-to-severe jaw muscle pain	15 enrolled; 10 completed	BoNT-A	25 U per temporalis + 50 U per masseter; 3 injection sites per muscle; 0.2 cm^3^/site	Bilateral masseter + temporalis	Placebo; crossover at 16 weeks	Baseline; 8, 16 and 24 weeks	VAS pain intensity/unpleasantness; maximum interincisal opening; muscle tenderness	No significant benefit for pain or tenderness; maximum opening was significantly lower during the BoNT-A arm. Findings did not support BoNT-A for this population.
Guarda-Nardini et al. (2008)^14^	Randomized double-blind placebo-controlled pilot	Adults with bruxism plus explicit masticatory myofascial pain; bruxism treated as a comorbidity rather than sole diagnosis	20	Botox® (onabotulinumtoxinA)	Per original pilot protocol	Masticatory muscles	Saline placebo	1 week; 1 and 6 months	Pain; mandibular movement; function/tolerance	Clinical improvement reported; tolerance assessed. Small pilot with mixed phenotype.
Ernberg et al. (2011)^15^	Randomized, double-blind, multicenter crossover trial	Persistent myofascial TMD pain refractory to conventional treatment	21	BoNT-A	Per trial protocol	Masticatory muscles	Placebo	Crossover follow-up	Pain; clinical response	No clear clinically relevant superiority over placebo; long-term safety mapping limited.
Guarda-Nardini et al. (2012)^16^	Randomized controlled comparative trial	RDC/TMD-defined myofascial pain of jaw muscles	30	BoNT-A	Single-session injection	Jaw muscles	Fascial Manipulation	End of treatment; 3 months	VAS pain; mandibular range of motion	Both interventions improved pain; differences were not clinically important at 3 months. No placebo arm.
De Carli et al. (2016)^17^	Randomized clinical trial	Patients with TMD-associated myofascial pain	15	BoNT-A	30 U at first session + 15 U after 15 days	Masticatory muscles; exact muscle distribution not specified in the abstract	Low-level GaAlAs laser therapy	Serial assessments through 30 days	VAS pain; mouth opening	Both therapies reduced pain; laser produced a faster response, whereas BoNT-A reached a similar reduction later. Neither treatment significantly improved mouth opening.
Chaurand et al. (2017)^18^	Prospective within-subject comparative clinical study	Masseter and temporalis myofascial pain associated with TMD	11	BoNT-A	Injection after 1 month of conservative therapy	Masseter + temporalis	Conservative-therapy phase	1 month after each phase	VAS; pressure algometry	Both approaches improved pain; BoNT-A produced additional improvement. Very small, nonparallel study.
Villa et al. (2019)^19^	Retrospective clinical study	RDC/TMD; all patients had myofascial pain	28	Botox® (onabotulinumtoxinA)	150 U total: 50 U per masseter (3 sites/side) + 25 U per temporalis (2 sites/side)	Bilateral masseter + temporalis	None	1 and 3 months	VAS; OHIP-14; TMJ-QoL	Pain and quality of life improved significantly at 1 and 3 months; no adverse effects reported.
De la Torre Canales et al. (2020)^22^	Randomized dose-ranging clinical trial	100 women with persistent masticatory myofascial pain	100	BoNT-A	Three dose groups (low/medium/high)	Masseter + temporalis	Saline placebo + oral appliance	Up to 24 weeks	Pain; pressure pain threshold; function	Pain improved over time without a clear dose-response advantage; masticatory performance, EMG, muscle thickness and mandibular bone volume were assessed, with dose-dependent adverse effects reported.
Montes-Carmona et al. (2020)^23^	Randomized clinical trial	DC/TMD-classified localized/referred masticatory myofascial pain	60	BoNT-A	Protocol following EMG evaluation	Masseter + temporalis + pterygoid muscles	Saline + lidocaine	7–180 days	Pain; mandibular movement; safety	BoNT-A reduced pain; response differed by pain phenotype; safety monitored to 6 months.
Miotto et al. (2021)^24^	Prospective controlled clinical study with random group allocation	Patients with chronic myofascial pain	40 (20/group)	BoNT-A	NR	Masticatory muscles	Counseling/self-care + Michigan-type occlusal splint	30 days	VAS pain; OHIP-14	Pain decreased and QoL improved in both groups; social disability and handicap domains favored conservative management.
Ângelo et al. (2026)^25^	Retrospective observational study	Adults with DC/TMD-confirmed myogenous TMD refractory to ≥3 months of conservative treatment	98	IncobotulinumtoxinA	Standardized Ângelo Botulinum Toxin Protocol®	Masticatory muscles	None	≥12 months	Myalgia severity; VAS orofacial pain; maximum mouth opening	Myalgia and pain significantly decreased at 12 months; mouth opening remained stable; 79.6% met favorable-outcome criteria; no complications were observed.
Kim et al. (2023)^26^	Randomized double-blind placebo-controlled pilot	Myogenous TMD with masticatory muscle pain and headache	21	BoNT/A	Tender-site injection protocol	Masticatory and adjacent cervical muscles	Saline placebo	4, 8 and 12 weeks	Orofacial pain; headache; tender points; mouth opening	Pain/headache outcomes improved; mouth-opening differences were limited; short-term adverse events monitored.
Reeve et al. (2024)^27^	Multicenter randomized double-blind placebo-controlled trial	DC/TMD-defined myofascial pain disorder	75	OnabotulinumtoxinA	Per trial protocol	Masseter/temporalis protocol	Saline placebo	1, 2 and 3 months	VAS; maximal incisal opening; JFLS; SF-36	Between-group pain differences were not statistically significant; short-term safety monitored.
Sitnikova et al. (2024)^28^	Randomized placebo-controlled crossover trial	Persistent TMD-related myofascial pain; DC/TMD algorithms	66	BoNT-A	50 U total	Masseter + temporalis	Saline placebo	2, 11 and 16 weeks	Pain; disability; EMG; bite force	Both BoNT-A and saline improved pain with no clear between-group superiority; BoNT-A significantly reduced muscle performance.
Shabaan et al. (2025)^29^	Prospective randomized clinical trial	Myofascial pain with masseter trigger points	42	OnabotulinumtoxinA (Botox®)	100-U vial reconstituted; 0.1 mL per trigger point	Masseter; ultrasound-guided intraoral vs extraoral approach	Intraoral vs extraoral BoNT-A technique	1, 3 and 6 months	VAS; maximum mouth opening; OHIP-14	Intraoral technique showed lower pain and better QoL than extraoral technique; no placebo group.
Park et al. (2025)^20^	Pilot prospective pre–post study	15 patients with myogenic TMD; mean age 51.42 years	15	BoNT-A	30 U masseter + 20 U temporalis	Masseter + temporalis	None	2, 4, 8 and 12 weeks	VAS; ultrasound muscle thickness; mouth opening	Rapid muscle thinning occurred within 2 weeks and persisted to 12 weeks; pain reduction was modest and not consistently significant, and pain and atrophy did not consistently correlate.
Henien et al. (2025)^30^	Clinical cohort/mixed-methods analysis	TMD-myofascial pain cohort	149	BoNT-A	Masseteric clinical protocol	Masseter	None	∼6 weeks primary reassessment	VAS; quality of life; symptom resolution	Pain and QoL improved; 21% complete symptom resolution reported; repeat-treatment exposure occurred in a subset.
Macedo de Sousa et al. (2025)^31^	Prospective single-arm pilot	Myofascial/myogenic TMD	25	BoNT-A	Single bilateral 50-U treatment	Masticatory muscles	None	6 and 12 weeks	VAS; Chronic Pain Index; JFLS-8; OHIP-14	Pain, function and QoL improved; only short-term safety data were available.
van Soest et al. (2025)^21^	Prospective observational cohort	DC/TMD-defined TMD-related myalgia	35	Xeomin® (incobotulinumtoxinA)	100 U total in 4 cc saline	Bilateral masseter + temporalis	None	1, 3 and 6 months	VAS; EQ-5D-3L; OHIP-14; MFIQ; mouth opening	Pain, health status and oral-health QoL improved; mandibular function and mouth opening did not significantly improve.
Lennartsson et al. (2025)^32^	Randomized, double-blind, placebo-controlled multicenter parallel-group trial	Adults ≥18 years with DC/TMD-diagnosed chronic orofacial myalgia persisting >6 months despite conservative treatment	72 enrolled; 69 completed	BoNT-A	Two BoNT-A injections separated by 3 months	Masticatory muscles	Saline placebo	1, 3, 4, 6 and 9 months	VAS pain intensity; longer-term clinical response	Pain decreased significantly from baseline in both groups, but BoNT-A provided no additional pain relief compared with saline at any follow-up.

**Abbreviations:** BoNT-A, botulinum toxin type A; DC/TMD, Diagnostic Criteria for Temporomandibular Disorders; EMG, electromyography; JFLS, Jaw Functional Limitation Scale; MFIQ, Mandibular Function Impairment Questionnaire; NR, not reported/insufficiently documented in the currently available source set; OHIP-14, Oral Health Impact Profile-14; QoL, quality of life; RDC/TMD, Research Diagnostic Criteria for Temporomandibular Disorders; VAS, visual analogue scale.

### 3.3 BoNT-A injection protocols

Substantial variability was observed in BoNT-A administration protocols. Differences involved toxin formulation, total dose, dose distribution between muscles, number of injection sites, muscles targeted, localization techniques, and treatment frequency. ^21^The masseter and temporalis were the most consistently targeted muscles, either alone or in combination. Some protocols additionally included other masticatory muscles according to the distribution of pain or muscular involvement. Villa et al. administered onabotulinumtoxinA (Botox®) bilaterally to the masseter and temporalis muscles, with a total dose of 150 U: 50 U per masseter distributed across three injection sites and 25 U per temporalis distributed across two sites. In contrast, van Soest et al. used incobotulinumtoxinA (Xeomin®), administering a total dose of 100 U to the bilateral masseter and temporalis muscles. Park et al. used a lower-dose protocol involving 30 U in the masseter and 20 U in the temporalis and monitored the resulting changes in muscle morphology using ultrasonography.^23^ Dose-ranging approaches were also identified among the randomized trials, allowing assessment of whether increasing BoNT-A exposure resulted in greater analgesic efficacy or increased physiological effects. However, the available evidence did not reveal a sufficiently consistent dose-response relationship to identify an optimal therapeutic dose. Overall, the marked variability in formulation, dose, muscle selection, injection sites, and localization methods prevented identification of a standardized BoNT-A injection protocol for myogenic TMD.^24^

### 3.4 Pain-related outcomes

Pain intensity was the most frequently investigated clinical outcome. Most observational studies and several controlled trials reported reductions in pain following BoNT-A injection; however, the magnitude of improvement and superiority over comparator interventions were inconsistent. Villa et al. observed significant reductions in visual analogue scale pain scores at both 1 and 3 months following BoNT-A administration.^19^ These improvements occurred concurrently with improvements in oral health-related quality of life. Similarly, van Soest et al. reported a clinically relevant reduction in pain at 1 month that was maintained during subsequent follow-up. In contrast, Park et al. observed relatively modest reductions in pain following treatment,^20^ with changes that were not consistently statistically significant across follow-up assessments despite clear ultrasonographic evidence of reduced muscle thickness. Importantly, placebo-controlled randomized trials yielded less consistent results than uncontrolled observational studies. Several trials reported improvements from baseline following BoNT-A administration but failed to demonstrate clear superiority over saline placebo across all follow-up periods. Other randomized studies reported greater analgesic effects of BoNT-A in selected patient groups or at specific follow-up intervals.Taken together, these findings suggest that within-group pain reduction following BoNT-.^26^ A is frequently observed, whereas the magnitude of the specific treatment effect beyond placebo or active conservative treatment remains less consistent. This distinction was particularly evident in studies using placebo-controlled designs and represents an important source of divergence within the available evidence.

### 3.5 Functional and patient-reported outcomes

Functional outcomes were less consistently evaluated than pain and demonstrated variable responses to BoNT-A treatment. Several studies assessed maximum mouth opening, mandibular mobility, jaw functional limitation, or masticatory performance. Improvements in pain did not consistently translate into measurable improvements in mandibular function. This discrepancy was particularly evident in the prospective cohort of van Soest et al. Although pain intensity, general health status, and oral health-related quality of life improved following BoNT-A administration, no significant improvement was observed in mandibular functional impairment or active and passive mouth opening.^27^ Quality-of-life outcomes generally showed more favorable trends. Villa et al. reported significant improvements in both OHIP-14 and TMJ-specific quality-of-life scores at 1 and 3 months.^19^ Van Soest et al. similarly found improvements in EQ-5D-3L and OHIP-14 scores.^21^ Collectively, the available evidence indicates that pain relief and patient-perceived improvement may occur without proportional recovery of objective mandibular function, suggesting that analgesic, functional, and patient-reported outcomes should be considered as distinct therapeutic domains.

### 3.6 Physiological effects and safety

Safety reporting varied considerably across studies. Conventional adverse events were generally uncommon; however, studies incorporating objective physiological measurements demonstrated that BoNT-A can produce measurable changes in masticatory muscle structure and function.^28^

Park et al. demonstrated a rapid reduction in masseter and temporalis muscle thickness following BoNT-A administration.^20^ Ultrasonographic changes were detectable within approximately 2 weeks and persisted during the 12-week observation period. Importantly, changes in muscle thickness did not consistently correlate with changes in pain intensity. This finding suggests that the biological effect of BoNT-A on muscle tissue may be substantially greater than would be inferred from pain outcomes alone.^29^ Other included clinical trials evaluated electromyographic activity, bite force, masticatory performance, and muscle thickness following BoNT-A administration. Collectively, these studies demonstrated temporary reductions in muscle activity and functional performance after treatment. Dose-ranging investigations additionally raised concerns regarding greater physiological effects with higher toxin exposure. Conversely, conventional clinical adverse events were infrequently reported in several observational studies. Villa et al., for example, reported no treatment-related adverse effects in their cohort. The available evidence therefore indicates an important distinction between reported adverse events and measurable physiological consequences.^30^ Absence of clinically reported complications should not necessarily be interpreted as absence of biological effects on the masticatory system.

### 3.7 Relationship between muscle changes and clinical response

An important finding emerging from the more recent evidence was the apparent dissociation between the neuromuscular effects of BoNT-A and its analgesic response.^31^ BoNT-A predictably reduces neuromuscular activity, and several studies documented reductions in muscle thickness, electromyographic activity, bite force, or masticatory performance. Nevertheless, these changes were not consistently associated with proportional reductions in pain. The prospective ultrasonographic study by Park et al. illustrates this finding particularly clearly: substantial reductions in muscle thickness were observed during follow-up, whereas pain reduction was comparatively modest and no consistent relationship between the two outcomes was demonstrated. Similarly, evidence from randomized trials evaluating muscle performance suggests that pharmacological weakening of the masticatory musculature should not be considered synonymous with therapeutic pain control.^32^ These findings indicate that muscle paralysis or atrophy and analgesia may represent partially independent consequences of BoNT-A administration.

### 3.8 Methodological quality and risk of bias

Methodological quality varied substantially across the included studies. Among randomized trials, the most frequent concerns involved incomplete reporting of randomization and allocation concealment in earlier studies, inability to blind participants or clinicians when markedly different active interventions were compared, and reliance on subjective patient-reported pain outcomes. More recent multicenter placebo-controlled trials generally showed stronger methodological safeguards.

The non-randomized evidence was predominantly judged to have serious risk of bias, primarily because of uncontrolled confounding, absence of concurrent comparator groups, participant selection, and confounding by indication. These limitations were particularly relevant to retrospective cohorts and single-arm pre–post studies, in which post-treatment improvement could not be unequivocally attributed to BoNT-A. Overall, favorable findings were more frequent among uncontrolled studies, whereas randomized placebo-controlled trials produced more heterogeneous treatment effects. Domain-level and overall risk-of-bias judgments for the randomized and non-randomized studies are presented in Table 3.

**Table 3:** Risk-of-bias assessment of the included clinical studies.

A. Randomized clinical trials assessed using Cochrane RoB 2							
Study	D1	D2	D3	D4	D5	Overall risk of bias	Main concern
Nixdorf et al. (2002)^13^	Some concerns	Low	High	Low	Some concerns	High	Substantial attrition in the crossover trial (15 enrolled; 10 completed) and limited reporting of allocation methods.
Guarda-Nardini et al. (2008)^14^	Some concerns	Low	Low	Low	Some concerns	Some concerns	Small pilot trial; insufficient detail on sequence generation/allocation concealment and selective-reporting safeguards.
Ernberg et al. (2011)^15^	Some concerns	Low	Low	Low	Some concerns	Some concerns	Multicenter double-blind crossover design, but limited reporting of randomization/concealment and prespecified reporting.
Guarda-Nardini et al. (2012)^16^	Some concerns	Some concerns	Low	High	Some concerns	High	Active interventions could not be blinded; subjective pain outcomes were vulnerable to knowledge of treatment assignment.
De Carli et al. (2016)^17^	Some concerns	Some concerns	Low	High	Some concerns	High	Open comparison of laser versus BoNT-A with subjective VAS outcomes; blinding and allocation concealment were not described in the indexed primary report.
De la Torre Canales et al. (2020)^22^	Low	Low	Low	Some concerns	Some concerns	Some concerns	Dose-ranging randomized design; subjective outcomes and multiple treatment arms create some concern regarding measurement/report selection.
Montes-Carmona et al. (2020)^23^	Some concerns	Low	Low	Some concerns	Some concerns	Some concerns	Randomized comparative design, but blinding and prespecified outcome-reporting procedures were not fully clear from available reporting.
Miotto et al. (2021)^24^	Some concerns	Some concerns	Low	High	Some concerns	High	Participants were randomly divided, but the active interventions could not be blinded and primary outcomes were patient-reported pain/QoL.
Kim et al. (2023)^26^	Low	Low	Low	Low	Some concerns	Some concerns	Double-blind placebo-controlled pilot with small sample; selective-reporting safeguards and precision were limited.
Reeve et al. (2024)^27^	Low	Low	Low	Low	Low	Low	Multicenter randomized double-blind placebo-controlled trial with strong safeguards against performance and measurement bias.
Sitnikova et al. (2024)^28^	Some concerns	Low	Low	Low	Some concerns	Some concerns	Placebo-controlled crossover design; potential period/carryover and selective-reporting issues require consideration.
Shabaan et al. (2025)^29^	Some concerns	High	Low	High	Some concerns	High	Comparison of intraoral versus extraoral techniques precluded participant/operator blinding; subjective outcomes and reporting transparency raise concern.
Lennartsson et al. (2025)^32^	Low	Low	Low	Low	Low	Low	Randomized double-blind placebo-controlled multicenter trial with extended follow-up and robust reporting.

B. Non-randomized studies assessed using ROBINS-I									
Study	D1	D2	D3	D4	D5	D6	D7	Overall risk of bias	Main concern
Chaurand et al. (2017)^18^	Serious	Moderate	Low	Moderate	Low	Moderate	Moderate	Serious	Small within-subject nonparallel study; strong potential for time effects, co-intervention, regression to the mean, and uncontrolled confounding.
Villa et al. (2019)^19^	Serious	Moderate	Low	Moderate	Low	Moderate	Moderate	Serious	Retrospective uncontrolled design; confounding and selection of patients receiving BoNT-A limit causal inference.
Park et al. (2025)^20^	Serious	Moderate	Low	Moderate	Low	Low	Moderate	Serious	Single-arm pilot without comparator; natural history, regression to the mean and concomitant factors cannot be separated from treatment effects.
Henien et al. (2025)^30^	Serious	Moderate	Low	Moderate	Moderate	Moderate	Moderate	Serious	Real-world uncontrolled cohort with repeat treatment in a subset and potential confounding by indication.
Macedo de Sousa et al. (2025)^31^	Serious	Moderate	Low	Moderate	Low	Moderate	Moderate	Serious	Single-arm prospective pilot; no concurrent control and small sample substantially limit causal attribution.
van Soest et al. (2025)^21^	Serious	Moderate	Low	Moderate	Low	Low	Moderate	Serious	Prospective pragmatic cohort without comparator; previous BoNT-A treatment was permitted, increasing confounding and expectation effects.
Ângelo et al. (2026)^25^	Serious	Moderate	Low	Moderate	Moderate	Moderate	Moderate	Serious	Retrospective observational design in refractory patients without a control group; confounding by indication and selection remain substantial.

**RoB 2 domains:** D1, randomization process; D2, deviations from intended interventions; D3, missing outcome data; D4, outcome measurement; D5, selection of the reported result. Judgments: Low risk, Some concerns, High risk.

**ROBINS-I domains:** D1, confounding; D2, participant selection; D3, classification of interventions; D4, deviations from intended interventions; D5, missing data; D6, measurement of outcomes; D7, selection of the reported result. Judgments: Low, Moderate, Serious, Critical risk of bias.

**Interpretive note:** Risk-of-bias judgments were made conservatively from the reporting available in the primary studies and source set. Earlier or inaccessible reports with insufficient methodological detail were not assumed to have low risk; lack of reporting was reflected as ‘Some concerns' or higher where appropriate.

### 3.9 Sources of clinical and methodological heterogeneity

Several sources of heterogeneity were identified across the evidence base. These included differences in diagnostic criteria and TMD phenotype; previous failure of conservative therapy; presence of comorbid bruxism or headache; BoNT-A formulation; total dose and dose per muscle; muscles selected for injection; number and localization of injection sites; use of anatomical, palpation-based, electromyographic, or ultrasound-guided localization; comparator intervention; outcome instruments; and follow-up duration.^25^ Outcome assessment was particularly heterogeneous. Pain was measured using different scales and time points, whereas mandibular function, quality of life, pressure pain threshold, muscle morphology, electromyographic activity, bite force, and bone-related outcomes were evaluated only in subsets of studies. This heterogeneity limited direct comparison across studies and precluded identification of a single injection protocol associated with consistently superior clinical outcomes.

### 3.10 Evidence gaps

Several clinically relevant evidence gaps emerged from the review. First, there was no consistently applied BoNT-A protocol regarding formulation, total dose, dose distribution, target muscles, or injection technique. Second, diagnostic heterogeneity remained substantial, particularly between older studies using RDC/TMD or clinical definitions and more recent investigations applying DC/TMD criteria. Third, many studies prioritized short-term pain reduction, whereas objective functional and physiological outcomes were less frequently assessed. Fourth, longer-term safety remains insufficiently characterized, particularly regarding repeated injections, sustained reductions in muscle mass or performance, and potential effects on mandibular bone.34 Finally, the discordance between favorable findings in uncontrolled studies and the less consistent superiority observed in placebo-controlled trials indicates that the magnitude of the BoNT-A-specific analgesic effect remains uncertain. Accordingly, the current evidence supports the presence of a biological and clinically measurable effect of BoNT-A on the masticatory system, but does not yet establish a standardized treatment protocol or a consistently superior therapeutic effect across patients with myogenic TMD.

## 4 Discussion

This scoping review mapped the clinical evidence regarding botulinum toxin type A (BoNT-A) for myogenic temporomandibular disorders, with particular emphasis on injection protocols, clinical outcomes, functional and physiological effects, safety, and methodological limitations.^33^ In relation to the primary review objective, the evidence indicates that BoNT-A produces measurable biological effects on the masticatory musculature and is frequently associated with reductions in pain and improvements in patient-reported outcomes; however, these effects do not translate into consistent superiority over placebo or conservative treatments across controlled trials. In relation to the secondary objective, substantial heterogeneity in patient selection, diagnostic criteria, toxin formulation, dose, injection technique, outcome assessment, and follow-up currently prevents the definition of a standardized therapeutic protocol.^34^ These two findings are clinically interconnected: uncertainty regarding the magnitude of the treatment-specific analgesic effect occurs in parallel with uncertainty regarding which patients, doses, and injection strategies provide the most favorable benefit-risk balance.

A particularly relevant finding was the apparent discrepancy between observational and randomized evidence. Uncontrolled and observational studies generally reported favorable reductions in pain following BoNT-A administration.^35^ Villa et al., for example,^19^ observed significant reductions in pain together with improvements in OHIP-14 and TMJ-specific quality-of-life scores following bilateral masseter and temporalis injections. Similarly, van Soest et al. reported clinically^21^ relevant pain reduction and improvement in health- and oral health-related quality of life after incobotulinumtoxinA treatment. In contrast, several placebo-controlled trials demonstrated improvement in both BoNT-A and control groups, with smaller or nonsignificant between-group differences. This distinction is clinically important because improvement from baseline alone cannot establish a toxin-specific therapeutic effect.^36^

Several factors may contribute to this discrepancy. Myogenic TMD is characterized by fluctuations in symptom intensity, and clinical improvement may reflect regression to the mean, natural symptom variation, contextual effects, concomitant treatment, or placebo responses in addition to the pharmacological action of BoNT-A.^37^ Injection procedures themselves may also produce nonspecific effects through local needling and patient expectations. Accordingly, favorable pre-post changes in uncontrolled studies should not be interpreted as equivalent to a treatment effect demonstrated by between-group comparisons in randomized placebo-controlled trials. This distinction directly affects clinical interpretation: the available literature supports the possibility of symptomatic improvement after BoNT-A, but the magnitude of benefit specifically attributable to the toxin remains less certain.

### 4.1 Analgesic effects and clinical relevance

Pain reduction remains the principal rationale for the clinical use of BoNT-A in myogenic TMD. Its mechanism of action is traditionally attributed to inhibition of acetylcholine release at the neuromuscular junction, resulting in temporary chemodenervation and decreased muscle contractility. Additional antinociceptive mechanisms have been proposed, including modulation of peripheral release of pain-related neuropeptides and neurotransmitters.^38^ Nevertheless, the clinical findings mapped in the present review suggest that neuromuscular inhibition and analgesia should not be considered equivalent therapeutic phenomena. This distinction is illustrated by Park et al., who demonstrated measurable reductions in masseter and temporalis thickness after BoNT-A injection while observing comparatively modest changes in pain.^20^ Changes in muscle thickness did not consistently correlate with changes in pain intensity. Thus, a strong biological effect on muscle tissue does not necessarily predict a proportional analgesic response. This observation is consistent with the absence of a reproducible dose-response relationship for analgesia and helps explain why increasing toxin exposure may intensify physiological effects without generating proportionally greater clinical benefit. Consequently, the therapeutic objective should not be maximal reduction of masticatory muscle activity, but rather the lowest effective exposure capable of achieving clinically meaningful symptom control while preserving function.^39^

### 4.2 Pain relief does not necessarily imply functional recovery

Another important finding was the inconsistent relationship between pain reduction and mandibular function. Patient-reported pain and quality-of-life measures frequently improved after BoNT-A administration, whereas objective or semi-objective functional measures demonstrated less consistent changes. Van Soest et al. provide a representative example.^21^ Despite improvements in pain, EQ-5D-3L, and OHIP-14 outcomes, significant improvements in mandibular functional impairment and active or passive mouth opening were not observed. Similarly, other studies assessing maximum mouth opening, bite force, electromyographic activity, or masticatory performance suggest that symptom improvement cannot automatically be interpreted as restoration of normal masticatory function. This finding addresses the review objective of examining clinical and physiological outcomes simultaneously and has direct practical implications: treatment success in myogenic TMD should be multidimensional and include pain, mandibular function, functional limitation, oral health-related quality of life, and potential treatment-related impairment. Future clinical trials should therefore avoid defining efficacy exclusively through changes in pain scores.

### 4.3 Injection protocol heterogeneity and the absence of an optimal dose

One of the clearest findings of this review was the lack of standardization in BoNT-A administration. Considerable differences were observed in toxin formulation, total dose, dose per muscle, number of injection points, muscles treated, localization techniques, and frequency of administration.^40^

The masseter and temporalis were the most frequently targeted muscles, but even within this relatively consistent anatomical pattern, substantial dose variation occurred. Villa et al. administered 150 U of onabotulinumtoxinA across the bilateral masseter and temporalis muscles, whereas van Soest et al. administered a total of 100 U of incobotulinumtoxinA. Park et al. employed a lower-dose protocol involving 30 U in the masseter and 20 U in the temporalis. Importantly, BoNT-A preparations cannot necessarily be considered interchangeable on a unit-for-unit basis. Therefore, reporting only the numerical number of units without clearly identifying the formulation, dilution, injection distribution, and anatomical targets limits reproducibility and comparison between studies. The available evidence does not support a single optimal dose.^41^ More importantly, the absence of a clear dose-response relationship for analgesia, together with evidence of dose-dependent physiological effects, argues against assuming that higher doses are therapeutically preferable. This protocol heterogeneity directly answers the secondary review question by identifying a major barrier to standardization and to evidence-based dose selection. Dose optimization should therefore balance potential analgesic benefit against preservation of muscle function.

### 4.4 Safety: beyond conventional adverse events

The safety profile of BoNT-A in myogenic TMD deserves particular attention. Most studies reported few serious conventional adverse events, which could suggest that the intervention is generally well tolerated. However, such reporting provides an incomplete assessment of safety because the pharmacological effect of BoNT-A inherently alters neuromuscular function.^33^, ^42^ Objective investigations have demonstrated reductions in muscle thickness, electromyographic activity, bite force, and masticatory performance after injection. Park et al. demonstrated ultrasonographically detectable muscle thinning within weeks of treatment, persisting throughout the 12-week follow-up.^20^ These findings are especially relevant because the magnitude of muscle change was not consistently associated with pain improvement. Therefore, absence of reported adverse events should not be equated with absence of physiological consequences. This distinction is particularly relevant for repeated treatment. A transient reduction in muscle activity following a single injection may be clinically acceptable in selected patients, but the cumulative implications of repeated chemodenervation remain insufficiently characterized.^26^, ^27^ Potential consequences involving sustained muscle atrophy, impaired masticatory performance, changes in bite force, and possible mandibular bone adaptation warrant further longitudinal investigation. From a benefit-risk perspective, these physiological effects are clinically meaningful even when they are not reported as conventional adverse events. Safety assessment in future trials should therefore extend beyond spontaneously reported adverse events and systematically include functional and structural outcomes.^43^

### 4.5 Patient selection May Be more important than dose escalation

The heterogeneity of treatment response suggests that patient selection may be a major determinant of therapeutic benefit. Myogenic TMD is not a uniform disorder. Patients may differ in pain duration, muscular involvement, central sensitization, psychosocial burden, bruxism, headache comorbidity, previous treatment exposure, and the relative contribution of peripheral muscle activity to their pain experience. BoNT-A may therefore be more biologically plausible in selected phenotypes characterized by a substantial peripheral muscular component than in patients whose persistent pain is predominantly maintained by central or psychosocial mechanisms. This interpretation remains a hypothesis generated by the heterogeneity mapped in the present review rather than a validated treatment-selection rule. It may nevertheless partially explain why studies enrolling refractory patients or different TMD phenotypes have produced heterogeneous responses despite apparently similar injection protocols. The available evidence consequently does not support indiscriminate use of BoNT-A across all patients with myogenic TMD. Future investigations should focus on identifying reproducible clinical characteristics associated with treatment response and determining whether specific phenotypes derive greater net benefit.

### 4.6 Position of BoNT-A within the therapeutic strategy

The findings of this review should be interpreted within the broader principle that management of myogenic TMD is predominantly conservative.^8^, ^9^ Education, behavioral modification, therapeutic exercise, physiotherapy, pharmacological management when appropriate, and occlusal appliances remain commonly used first-line strategies. The inconsistent superiority of BoNT-A over placebo or conservative comparators does not support its routine use as a universal first-line treatment for myogenic TMD. Instead, the available evidence is more compatible with a potential adjunctive role in carefully selected patients with persistent symptoms who have not achieved adequate improvement following appropriate conservative management.^44^ This positioning is also important from a benefit-risk perspective. Unlike reversible behavioral or physical interventions, BoNT-A intentionally reduces neuromuscular activity and may produce measurable structural and functional changes. The threshold for its use should therefore reflect symptom severity and persistence, prior response to conservative care, and the availability of less invasive therapeutic alternatives.

### 4.7 Comparison with previous evidence syntheses

Previous systematic reviews and meta-analyses have reached differing conclusions regarding the efficacy of BoNT-A for TMD-related muscular pain. Some analyses have suggested reductions in pain compared with placebo at selected follow-up intervals, whereas others have failed to demonstrate consistent superiority. A 2024 meta-analysis, for example, found that only a subset of the eligible randomized trials could be quantitatively pooled because of substantial clinical and methodological heterogeneity.

More recently, a 2026 systematic review focused specifically on duration of clinical effects and dosage parameters in myogenous TMD concluded that the available randomized evidence remains insufficient to support routine first-line use and emphasized substantial variability in treatment protocols. The present review is broadly concordant with those conclusions but extends previous syntheses by examining BoNT-A beyond analgesic efficacy alone.^42^ By simultaneously mapping injection protocols, patient-reported outcomes, mandibular function, muscle morphology, neuromuscular performance, safety signals, and methodological quality, our findings highlight an important clinical distinction: the magnitude of the biological effect on masticatory muscle may not parallel the magnitude of pain relief. Thus, the contribution of this scoping review is not a new pooled efficacy estimate, but a broader interpretation of how efficacy, function, physiological effects, and safety interact when judging the clinical role of BoNT-A.

### 4.8 Methodological implications and research priorities

Several methodological priorities emerge from the current evidence. Future trials should adopt standardized diagnostic criteria, preferably contemporary DC/TMD definitions, and clearly characterize the myogenic phenotype being treated. Studies should report the exact BoNT-A formulation, dilution, total dose, dose per muscle and per injection point, anatomical localization method, number of treatment sessions, and previous exposure to botulinum toxin.

A core outcome framework would also improve comparability. At minimum, future trials should assess pain intensity, clinically meaningful pain response, mandibular function, patient-reported quality of life, adverse events, and objective measures of masticatory muscle function. Where repeated or higher-dose treatment is investigated, muscle morphology, bite force, electromyographic activity, and potentially bone-related outcomes should be incorporated. 6, 7 Adequate follow-up is particularly important. Many existing studies focus on the first weeks or months after injection, whereas the consequences of repeated treatment cycles remain substantially less understood. Finally, sufficiently powered randomized placebo-controlled trials are required to identify treatment-response phenotypes and establish whether particular patient subgroups obtain clinically meaningful benefits that outweigh the physiological effects of treatment.

### 4.9 Strengths and limitations

This scoping review has several strengths. It provides a broad evaluation of BoNT-A in myogenic temporomandibular disorders by integrating evidence from randomized controlled trials and observational clinical studies and by examining not only pain reduction but also injection protocols, patient-reported outcomes, mandibular function, muscle morphology, neuromuscular performance, and safety.^25^ Particular attention was given to differences in diagnostic criteria, BoNT-A formulations, dosing regimens, target muscles, and follow-up periods. Furthermore, studies reporting nonsignificant or unfavorable findings were retained, allowing a more balanced interpretation of the available evidence and reducing the risk of selectively emphasizing positive treatment effects. Several limitations should nevertheless be acknowledged.^20^, ^29^, ^30^ The limitations with the greatest potential to materially affect interpretation are the clinical and methodological heterogeneity of the included evidence and the risk of bias in non-randomized or uncontrolled studies. Differences in diagnostic criteria, patient characteristics, BoNT-A formulations, total doses and dose distribution, muscles targeted, injection techniques, comparator interventions, outcome measures, and follow-up periods limited direct comparisons between studies and precluded identification of a standardized treatment protocol. Methodological quality and sample size also varied considerably. Several studies were pilot investigations or involved relatively small samples, whereas observational and uncontrolled studies were susceptible to selection bias, confounding, regression to the mean, and placebo or contextual effects. Consequently, improvements observed after treatment in uncontrolled studies cannot be interpreted as evidence of a BoNT-A-specific therapeutic effect. Outcome assessment was also insufficiently standardized. Pain intensity was the most frequently evaluated endpoint, but different scales, definitions of treatment response, and assessment time points were used. Functional outcomes, quality of life, electromyographic activity, bite force, muscle morphology, and bone-related outcomes were assessed less consistently, limiting comparison between analgesic and physiological responses.^12^, ^13^, ^14^.

Safety reporting represents an additional important limitation because conventional adverse-event reporting was more common than systematic assessment of objective physiological consequences of chemodenervation.^33^ Reductions in muscle thickness, electromyographic activity, bite force, or masticatory performance were evaluated in only a subset of studies, and their long-term clinical relevance, particularly after repeated BoNT-A treatment cycles, remains uncertain. Follow-up duration also varied substantially and was relatively short in several studies, limiting conclusions regarding durability of analgesic effects, recovery of neuromuscular function, cumulative effects of repeated injections, and possible long-term structural consequences. These limitations materially constrain benefit-risk interpretation. In contrast, the absence of a pooled effect estimate has a more limited implication because quantitative efficacy estimation was not the primary objective of this scoping review; rather, the review was designed to map the breadth, characteristics, heterogeneity, and gaps of the evidence.^38^ Quantitative synthesis would be appropriate only for sufficiently comparable subsets of randomized trials.^41^, ^43^ Accordingly, the conclusions of this review emphasize the consistency, uncertainty, and clinical implications of the available evidence rather than a single estimate of therapeutic effect.

### 4.10 Clinical interpretation

The available evidence suggests that BoNT-A should not be conceptualized simply as an analgesic intervention for myogenic TMD. It is a potent neuromuscular treatment that can reduce pain in some patients while simultaneously producing measurable changes in masticatory muscle structure and function. With respect to the primary review objective, the evidence supports possible symptomatic benefit but does not demonstrate consistent treatment-specific superiority across controlled trials. With respect to the secondary objective, heterogeneity in diagnosis, patient selection, formulation, dose, injection strategy, outcomes, and follow-up currently prevents protocol standardization. Current evidence does not establish that greater muscular inhibition produces greater analgesic benefit, nor does it identify a standardized dose or injection protocol associated with an optimal benefit-risk profile. The overall contribution of this review is therefore to show that analgesic response, neuromuscular effect, functional outcome, and safety should be evaluated as related but distinct domains. Until these uncertainties are resolved, BoNT-A appears more appropriately positioned as a selective adjunctive intervention for carefully diagnosed, persistent myogenic TMD rather than as a routine first-line therapy.

## 5 Conclusions

Botulinum toxin type A produces clinically and physiologically measurable effects in patients with myogenic temporomandibular disorders. Although reductions in pain and improvements in patient-reported outcomes have been frequently reported, particularly in observational studies, randomized placebo-controlled trials have demonstrated less consistent superiority. Consequently, improvement from baseline should not be interpreted as definitive evidence of a BoNT-A-specific analgesic effect.

A central finding of this review is that the neuromuscular effects of BoNT-A do not necessarily parallel its analgesic effects. Reductions in muscle thickness, electromyographic activity, bite force, and masticatory performance may occur without proportional pain relief. This dissociation is clinically relevant because it indicates that treatment success should be evaluated using multidimensional outcomes rather than pain intensity alone. Current evidence does not support a standardized BoNT-A protocol for myogenic TMD with respect to formulation, dose, target muscles, injection sites, or treatment frequency. Furthermore, the long-term consequences of repeated chemodenervation remain insufficiently characterized. BoNT-A should therefore not be considered a routine first-line intervention but may represent a selective adjunctive option for carefully diagnosed patients with persistent myogenic TMD who have not adequately responded to conservative management. Future adequately powered randomized trials using standardized DC/TMD diagnoses, clearly reported injection protocols, multidimensional outcomes, and longer follow-up are required to establish patient-selection criteria and an optimal benefit–risk profile.

## Author contributions

BVR contributed to the conception and design of the project, obtained full access to the data, and was primarily accountable for all aspects of the work, ensuring the integrity and accuracy of the research and the drafting of the manuscript. FC,MVS,VMC,LBF, MRT, LCH-B and BVR contributed with the revision of the available literature. FC,MVS,VMC,LBF, MRT, LCH-B and BVR contributed to the analysis, internal validity of the study, and initial drafting of the manuscript FC,MVS,VMC,LBF, MRT, LCH-B and BVR critically reviewed and edited the manuscript, and providing input toward the reporting of the data and its interpretation. All authors contributed to the article and approved the submitted version.

## Data availability

Not applicable.

## Ethical statement

Not required as it is a review of literature.

## Human subjects statement

This study is a scoping review of previously published studies and did not involve direct recruitment, intervention, or collection of identifiable data from human participants. Therefore, ethics committee approval and informed consent were not required.

## Animal subjects statement

No animals were directly involved in this study.

## Funding

No external funding was received for this research. There were no funding sources for the development of this research.

## Declaration of competing interest

The authors declare that they have no known competing financial interests or personal relationships that could have appeared to influence the work reported in this paper.

## Figures and Tables

**Table:** 

(BoNT-A)	Botulinum toxin type A
TMD	Temporomandibular Disorders
OHIP-14), EQ-5D	Oral Health Impact Profile

## Abbreviations

The following abbreviations are used in this manuscript:
